# Identification of Volatile Compounds and Selection of Discriminant Markers for Elephant Dung Coffee Using Static Headspace Gas Chromatography—Mass Spectrometry and Chemometrics

**DOI:** 10.3390/molecules23081910

**Published:** 2018-07-31

**Authors:** Poowadol Thammarat, Chadin Kulsing, Kanet Wongravee, Natchanun Leepipatpiboon, Thumnoon Nhujak

**Affiliations:** 1 Chromatographic Separation and Flavor Chemistry Research Unit, Department of Chemistry, Faculty of Science, Chulalongkorn University, Bangkok 10330, Thailand; poohach@hotmail.com (P.T.); ckulsing@gmail.com (C.K.); kanet.w@chula.ac.th (K.W.); 2 Center of Molecular Sensory Science, Faculty of Science, Chulalongkorn University, Bangkok 10330, Thailand; 3 Sensor Research Unit, Department of Chemistry, Faculty of Science, Chulalongkorn University, Bangkok 10330, Thailand; 4 Betagro Science Center Co., Ltd., Pathumthani 12120, Thailand

**Keywords:** elephant dung coffee, volatile compound, discriminant marker, SHS GC-MS, chemometrics, coffee authentication

## Abstract

Elephant dung coffee (Black Ivory Coffee) is a unique Thai coffee produced from Arabica coffee cherries consumed by Asian elephants and collected from their feces. In this work, elephant dung coffee and controls were analyzed using static headspace gas chromatography hyphenated with mass spectrometry (SHS GC-MS), and chemometric approaches were applied for multivariate analysis and the selection of marker compounds that are characteristic of the coffee. Seventy-eight volatile compounds belonging to 13 chemical classes were tentatively identified, including six alcohols, five aldehydes, one carboxylic acid, three esters, 17 furans, one furanone, 13 ketones, two oxazoles, four phenolic compounds, 14 pyrazines, one pyridine, eight pyrroles and three sulfur-containing compounds. Moreover, four potential discriminant markers of elephant dung coffee, including 3-methyl-1-butanol, 2-methyl-1-butanol, 2-furfurylfuran and 3-penten-2-one were established. The proposed method may be useful for elephant dung coffee authentication and quality control.

## 1. Introduction

Coffee is one of the most popular beverages worldwide because of its desirable taste and smell, as well as its desirable properties, such as providing refreshment and immune stimulation. Moreover, coffee is the most important agricultural product in some countries [1]. In addition to the normal coffee beans produced by the conventional approach [2], there are special types obtained from the digestive system of animals, including civet coffee (Kopi Luwak) and elephant dung coffee (Black Ivory Coffee). A few scientific studies on civet coffee have been reported [3,4,5], while there are no previous reports on elephant dung coffee. Black Ivory Coffee, or elephant dung coffee, is a brand of coffee exclusively provided by the Black Ivory Coffee Company in Thailand. This type of coffee derives from Arabica coffee (*Coffea arabica*) fruits collected from inside the feces of Asian elephants (*Elephas maximus*). Note that the elephants were fed with the coffee cherries combined with the other typical feeds, such as bananas, tamarinds and rice brans. No ethical concerns are related to the production of elephant dung coffee. The coffee cherries are initially digested and fermented with various other ingredients inside the elephant’s gastrointestinal tract for 12 to 70 h. After elephant excretion, the individual cherries are hand-picked by the elephant caregivers and are then washed and dried under sunlight with a certain percentage of moisture. The cherries are then hulled and sorted to obtain the perfect green beans [6]. The characteristic taste of Black Ivory Coffee has been described as “very smooth without the bitterness of regular coffee”, and at $1800 per kilogram, Black Ivory Coffee is among the world’s most expensive coffees. Moreover, it has a limited productivity of approximately 200 kg per year [7]. As mentioned above, the elephant dung coffee is unique and the production rate has been increasing the sources of which are provided by the original Chiang Rai Province and another new source at Surin Province. It is expected that if the consumption demand increases, this coffee could be made worldwide.

The chemical composition of coffee is very complex, consisting of a wide range of volatile and non-volatile compounds with various functionalities. The components of green coffee beans are mostly carbohydrates (mannan, arabinogalactan, cellulose and sugars), lipids, proteins, peptides, amino acids, alkaloids, organic acids and phenolic compounds. Many of these components are important precursors responsible for the coffee aroma after roasting [2,8,9,10]. Roasting is the process of converting green coffee beans to roasted coffee beans under heat treatment. This process involves several physical and chemical phenomena inside the coffee beans, such as thermal degradation, the Maillard reaction and Strecker degradation. During the roasting process, coffee beans release more than 900 volatile and semi-volatile compounds, such as acids, alcohols, aldehydes, esters, furans, indoles, ketones, phenolic compounds, pyrazines, pyridines, pyrroles and thiols [11,12,13,14,15]. These compounds can be correlated with coffee qualities such as aroma and flavor [16]. Furthermore, the process of coffee preparation, including the grinding and brewing method, also affects the aroma of the coffee brew [11,17].

Gas chromatography-mass spectrometry (GC-MS) is a powerful analytical technique that is widely used to profile volatile compounds in coffee because it can efficiently separate and identify compounds [11,12,18,19,20,21]. Coffee samples can be prepared by a solvent extraction method followed by direct liquid injection into the GC column, allowing the detection of volatile and semi-volatile compounds in coffee [12,17,22,23]. However, some volatile compounds may be lost during the sample preparation step. The ideal sample preparation method for the analysis of aroma compounds in coffee is a headspace approach that involves the sampling of the gas phase above the sample in a closed container. Headspace-solid phase microextraction (HS-SPME) with a specific fiber has been widely used to extract volatile compounds from coffee samples and is followed by thermal desorption at the GC injector [10,11,18,24]. Dynamic headspace (DHS) with a suitable adsorbent tube equipped with a thermal desorption unit (TDU) has also been reported for coffee analysis [20,21,25]. Alternatively, static headspace (SHS) sampling can be simply performed to extract volatile compounds from coffee samples without the use of specific solid-phase materials. By this method, all volatile compounds are introduced into the GC-MS system [19,22,26,27,28]. In some cases, gas chromatography-olfactometry (GC-O) with a sensory evaluation by panelists has been used for the identification of key odorants in coffee [29,30].

Since the chemical profile of coffee obtained with GC-MS is very complex, consisting of a large number of compounds, statistical and chemometric methods are necessary for data processing, including unsupervised pattern recognition (e.g., hierarchical cluster analysis (HCA) and principal component analysis (PCA)), supervised pattern recognition (e.g., linear discriminant analysis (LDA)), as well as a variable selection approach (e.g., one-way analysis of variance (ANOVA), T-statistics and iterative reformulation) [4,31,32,33,34].

In this study, the volatile compounds of elephant dung coffee (Black Ivory Coffee) were profiled using SHS GC−MS. According to the profile of the volatile compounds in the coffee brew, chemometric approaches were applied to identify the discriminant markers of elephant dung coffee.

## 2. Results and Discussion

### 2.1. Optimization of SHS GC-MS

**Figure 1:**
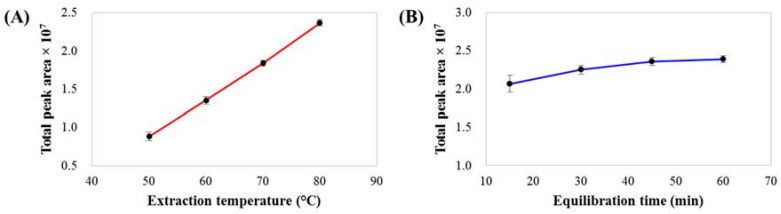
Effect of (**A**) extraction temperature and (**B**) equilibration time on total peak areas of volatile compounds from coffee brew samples.

The performance of static headspace extraction depends on several parameters, especially the extraction temperature and equilibration time. The effect of extraction temperature (50, 60, 70 and 80 °C, with an equilibration time of 60 min) on the total peak area of volatile compounds is shown in Figure 1A. Temperature is the most important parameter affecting the distribution coefficient (*K*), which is a thermodynamic constant involving the ratio of the concentration of compounds in the gas phase to that in the liquid phase at equilibrium. Extraction at higher temperatures can enhance the compound concentration in the gas phase, increasing the peak areas of volatile compounds and improving the overall performance of SHS. An extraction temperature of 80 °C was selected for further SHS analysis. Another important parameter is the equilibrium time. The effect of equilibrium time (15, 30, 45 and 60 min) on the total peak area of volatile compounds at an extraction temperature of 80 °C is shown in Figure 1B. Approximately 45 min is required to reach extraction equilibrium. However, to ensure SHS efficiency, an equilibration time of 60 min was chosen, which corresponded to the total GC-MS run time. Thus, this extraction process could be performed during the GC-MS analysis of the previous sample, as a result of which the total analysis time was not affected by the 60 min equilibration time. For the method precision evaluation, the analysis of a coffee brew sample was repeated by analyzing six vials of the same sample under the optimum SHS GC-MS conditions. The relative standard deviations (RSDs) of the percentages of the relative peak areas (%RPAs) of 78 tentative volatile compounds were in the range of 1.8–15% (the data is summarized in Table S1), indicating that the developed method has good precision.

### 2.2. GC-MS Analysis of Coffee Brew Samples and Compound Identification

**Figure 2:**
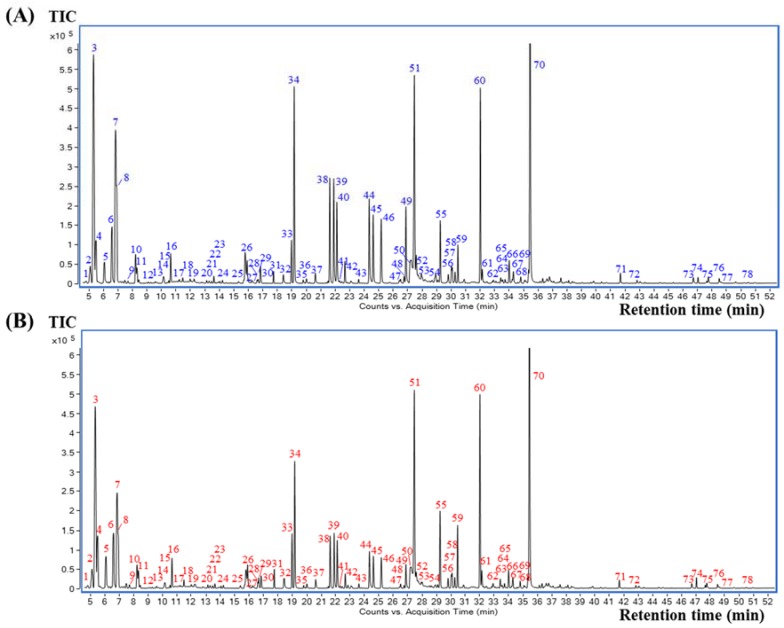
Representative total ion chromatograms (TICs) of coffee brew samples: (**A**) elephant dung coffee and (**B**) control (normal coffee beans collected from the same plantation, without the elephant digestion process).

The selected SHS method in the above section was applied prior to GC-MS analysis for all coffee brew samples. The volatile compounds were well separated on a polar DB-WAX capillary column, indicating that the column is suitable for coffee analysis. All the samples revealed similar volatile profiles. The representative total ion chromatograms (TICs) of the elephant dung coffee and control (normal coffee beans collected from the same plantation, without the elephant digestion process) are shown in Figure 2A,B, respectively. All peaks detected in the GC-MS chromatograms were identified according to a comparison of their mass spectra and the *LRI* with those contained in the NIST 14 database and in the literature. The criteria for compound identification required a mass spectrum matching score of ≥70 and an *LRI* difference of ≤20 units between the calculated *LRI* and the *LRI* from the database for the same stationary phase. Using these criteria, 78 volatile compounds were tentatively identified in both elephant dung coffee and the controls, as summarized in Table 1. Thirteen chemical classes were observed, including six alcohols, five aldehydes, one carboxylic acid, three esters, 17 furans, one furanone, 13 ketones, two oxazoles, four phenolic compounds, 14 pyrazines, one pyridine, eight pyrroles and three sulfur-containing compounds. These compounds have also been reported by several research groups that have analyzed coffee by HS GC-MS [11,17,19,20,21]. According to the %RPAs, the major compounds found in both sets of coffee samples were acetone, methylpyrazine, furfural, 5-methylfurfural and 2-furanmethanol, which are labelled as the peaks no. 3, 34, 51, 60 and 70, respectively.

### 2.3. Comparison of Volatile Compounds in Elephant Dung Coffee and Controls

**Figure 3:**
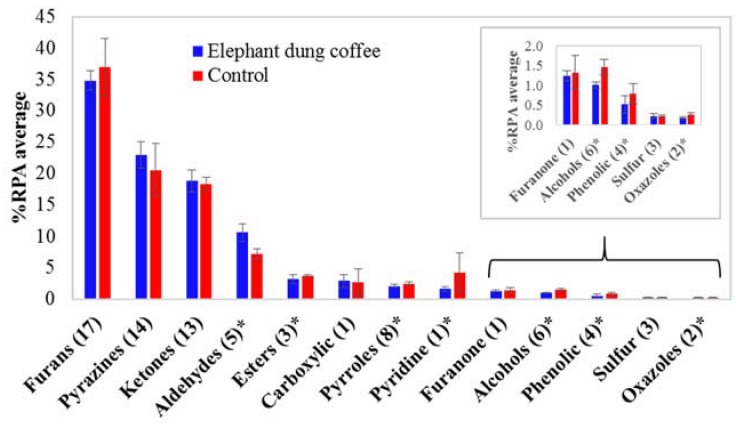
The average percentage of relative peak area (%RPA average) of each chemical class of elephant dung coffee brew and the control samples, (*n*) refers to the number of volatile compounds in each class, and * indicates the comparison between the two samples with *p*-value < 0.01.

As shown in Figure 2A,B, elephant dung coffee and the controls have a similar profile, presenting the same 78 tentative volatile compounds, which reveals that no unique compounds identify elephant dung coffee analyzed by using the present SHS GC-MS approach. Therefore, the quantitative aspects or comparisons of the compound peak areas are valuable. A comparison of the volatile compounds in each coffee sample was obtained according to the percentage of an individual peak area relative to the total peak area of all identified compounds in each chromatogram (%RPA). The fold-change of each compound was also calculated by comparing its %RPA average in elephant dung coffee and its %RPA average in the controls. The %RPA average and fold-change, as well as the T-statistics and *p*-value of each compound, are summarized in Table S2. The top five compounds with the highest %RPA average in elephant dung coffee are 2-furanmethanol (15.9%), acetone (12.6%), furfural (6.22%), methylpyrazine (5.28%) and 5-methylfurfural (5.21%). However, their values are close to those found for the controls. The results of fold-change analysis showed relatively lower amounts of 49 volatile compounds in the elephant dung coffee, whereas 29 compounds showed higher amounts in the elephant dung coffee than in the controls. According to the *p*-value < 0.01 criterion, the %RPA of 45 volatile compounds in elephant dung coffee and control are significantly different. In addition, a comparison of the %RPA average of each chemical class in elephant dung coffee and in the controls is shown in Figure 3. The top three classes with the highest %RPA in elephant dung coffee are furans (34.8%), pyrazines (22.9%) and ketones (18.8%). Nevertheless, they yield a *p*-value ≥ 0.01, which is insignificantly different from the control data. Regarding all the identified compounds, seven chemical classes are significantly different (*p*-value < 0.01), including aldehydes, esters, pyrroles, pyridines, alcohols, phenolic compounds and oxazoles.

### 2.4. Chemometric Approaches

#### 2.4.1. Hierarchical Cluster Analysis (HCA)

**Figure 4:**
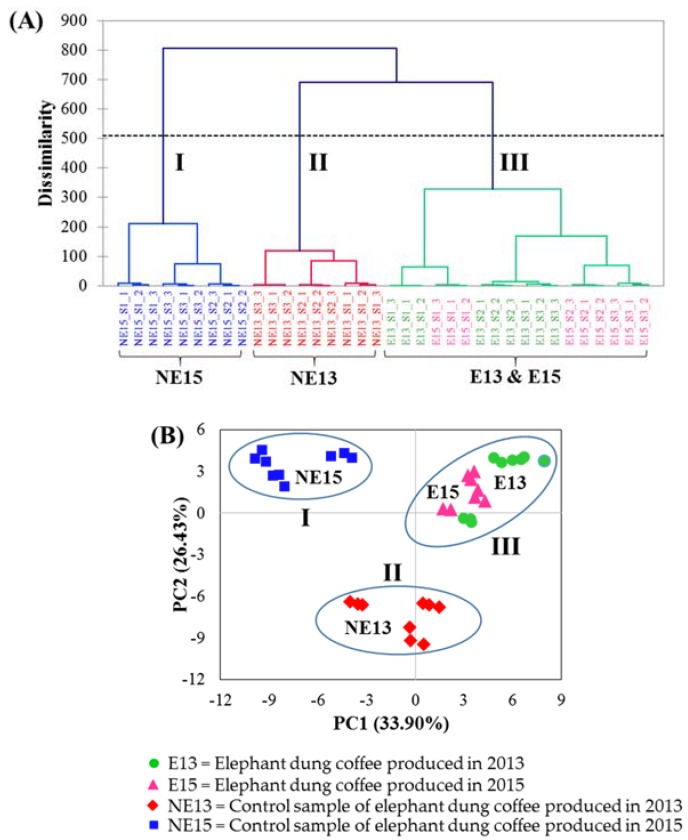
(**A**) The obtained dendrogram of HCA of coffee brew samples from different groups and (**B**) PCA score plot of 36 coffee brew samples with 78 variables.

HCA, an unsupervised pattern recognition method, was accomplished in order to evaluate the degrees of correlation among the coffee samples according to their standardized %RPA data. The analysis results were represented by different distances between the samples. The nearest distance shows the highest degree of correlation. As a result, samples in close proximity are considerably in the same cluster [34,36]. The results of HCA are presented as a dendrogram, shown in Figure 4A. By plotting the dissimilarity (y-axis) against the sample names (x-axis), three main clusters were achieved in the dendrogram. The results showed that clusters I and II comprise 9 control samples produced in 2015 (NE15) and 9 control samples produced in 2013 (NE13), respectively. Cluster III consists of both 9 elephant dung coffee samples produced in 2013 (E13) and 9 elephant dung coffee samples produced in 2015 (E15). This indicates that elephant dung coffees produced in different years have very similar volatile compositions.

#### 2.4.2. Principal Component Analysis (PCA)

PCA, a popular unsupervised pattern recognition technique utilized for the data visualization, was applied to evaluate whether the data of volatile compounds identified in all the coffee samples can be used to effectively differentiate between elephant dung coffee and control samples. The aim of PCA approach is to reduce the number of variables to a new and smaller number of variables, called principal components (PCs) [34,36]. PCA was performed according to the %RPA of 78 volatile compounds (variables) from 36 coffee brew samples as an input dataset for calculation, and the data were standardized before processing. The resulting PCA score plot is shown in Figure 4B. The first two PCs, including PC1 and PC2 were chosen in order to represent the data objects with the highest variation (33.90% and 26.43% of the variation). From the PCA score plot, coffee brew samples can be separated into three groups. Groups I and II are control samples of elephant dung coffee produced in 2015 (NE15) and 2013 (NE13), respectively. Group III is a combination of elephant dung coffee samples produced in 2013 (E13) and elephant dung coffee samples produced in 2015 (E15). The PCA grouping result was in agreement with that obtained using HCA, which confirms the reliability of the evaluation.

#### 2.4.3. Linear Discriminant Analysis (LDA)

LDA, a supervised pattern recognition method, was performed in order to generate a model to classify the studied coffee samples into different groups. The classifier model was constructed from a dataset of the known classes of samples. The model boundary between classes created using linear discriminant functions was accomplished in order to specify the directions in which the classes are best separated. The “leave-one-out” cross-validation (LOOCV) was operated for evaluating the predictive ability of the created model. The predictive ability is the percentage of samples belonging to the testing set correctly classified using the generated model [34,37]. The LDA classification outputs of all samples from the four groups (E13, E15, NE13 and NE15) in this study are shown in Table 2. The predictive ability of the constructed model was 89%, showing a satisfactory performance of this model for the class prediction of elephant dung coffee and controls according to the year of production. NE13 and NE15 achieved 100% correct classification, indicating the strong association between the volatile compound profiles and each coffee sample. For E13 and E15, the percentages of correct classifications were 67% and 89%, respectively. The incorrect classification of some samples of E13 and E15 may be caused by the similarity of the volatile compound profiles of E13 and E15. This implies that elephant dung coffees collected in 2013 and 2015 have very similar quality.

#### 2.4.4. Selection of Discriminant Markers for Elephant Dung Coffee

The selection of potential markers from tentative volatile compounds for elephant dung coffee was initially performed (according to their %RPAs) by using one-way ANOVA and T-statistics. To evaluate the differences in variation between the volatile compounds in elephant dung coffee and in the controls, one-way ANOVA was performed by using the criteria of a level of significance of 99% and a *p*-value of < 0.01. The *p*-values of the volatile compounds are summarized in Table S2. Based on this strategy, 45 volatile compounds were significantly different between elephant dung coffee and the controls and may be used as marker compounds. 

Next, T-statistics, a method for the significance testing of two populations, was applied to evaluate the volatile compounds in elephant dung coffee and in the controls. The *t*-value (or *t*-stat) was calculated for each compound according to the mean and standard deviation of the compound %RPAs in the two sample groups [34], and the results are summarized in Table S2. This method was considered since the interpretation of results is not complicate. The volatile compounds in elephant dung coffee and in the controls are significantly different if the calculated *t*-value is larger than the critical *t*-value. This approach resulted in the same number of (45) compounds as that obtained from one-way ANOVA. The level of difference is determined from the magnitude of the difference between the calculated *t*-value and the critical *t*-value. According to the *t*-values, the 8 top-ranking (10% of variables) compounds include 3-methyl-1-butanol (−18.5), 2-methyl-1-butanol (−14.6), 3-penten-2-one (−13.0), 2-furfurylfuran (−12.5), furfuryl methyl ether (−9.91), 2-methyl-2-cyclopenten-1-one (−9.18), 2-methylbutanal (8.96) and 3-methylbutanal (8.30), which were selected as a potentially good marker compounds for elephant dung coffee.

Furthermore, iterative reformulation, an effective approach for the proper validation of data and for the selection of potential markers [33], was performed. In this study, discriminant markers were chosen from the eight volatile compounds obtained from the *t*-stat selection above in 36 coffee brew samples. The numbers used for the training set, test set and iterations were 30, 6 and 100, respectively. The results in Table 3 show the frequency of selection for each volatile compound. A variable with a frequency of 100% was always selected in all 100 models. Therefore, 3-methyl-1-butanol, 2-methyl-1-butanol, 2-furfurylfuran and 3-penten-2-one were propo**s**ed as the potential markers of elephant dung coffee.

**Figure 5:**
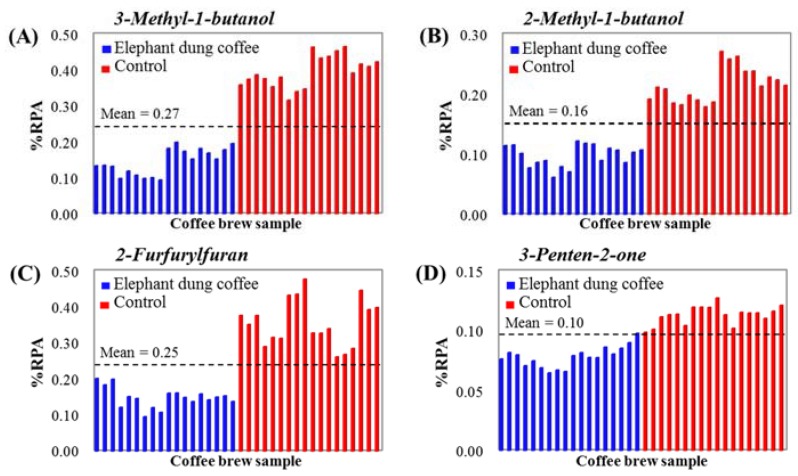
Distribution graphs of discriminant markers of elephant dung coffee: (**A**) 3-Methyl-1-butanol, (**B**) 2-Methyl-1-butanol, (**C**) 2-Furfurylfuran and (**D**) 3-Penten-2-one, compared to the control samples.

Finally, the confirmation of the proposed markers was performed by creating the distribution plots of their %RPAs in 36 samples, as shown in Figure 5A–D. Clear discrimination between elephant dung coffee and the controls is observed for all the selected markers. The %RPAs of compounds from elephant dung coffee are smaller than the mean value of all samples (represented as a dashed line), whereas the %RPAs of compounds from the controls are larger than the mean value of all samples. This may be caused by the effect of the digestion and fermentation of coffee cherries in the gastrointestinal tract of elephants during coffee bean production. As a result, 3-methyl-1-butanol, 2-methyl-1-butanol, 2-furfurylfuran and 3-penten-2-one were selected as the discriminant markers of elephant dung coffee.

## 3. Materials and Methods

### 3.1. Chemicals 

Two series of *n*-alkane standards, including the C_8_ to C_20_ (40 mg/L each, in hexane) and C_10_ to C_40_ (50 mg/L each, in heptane) series, and sodium chloride were purchased from Sigma-Aldrich (St. Louis, MO, USA). 

### 3.2. Coffee Bean Samples

Green (raw) beans of elephant dung coffee (Black Ivory Coffee) and controls (normal coffee beans collected from the same plantation, without the elephant digestion process) were obtained from Black Ivory Coffee Company (Chiang Rai, Thailand). These were Arabica (*Coffea arabica*) coffee beans cultivated in Chiang Mai, Thailand. The coffee bean samples were collected from two different harvesting years (2013 and 2015) and were randomly sampled three times for each year. The studied samples were divided into four subgroups, including elephant dung coffee produced in 2013 (E13), elephant dung coffee produced in 2015 (E15), controls for elephant dung coffee produced in 2013 (NE13) and controls for elephant dung coffee produced in 2015 (NE15). All green coffee samples were roasted under the same conditions. In this study, medium-roasted coffee beans were provided by a coffee factory using a conventional coffee roaster. Note that due to the novelty of the products launched in 2012, there was limited amount of the samples. Also, the samples were quickly sold out in 2012 and 2014. As a result, only the samples produced in 2013 and 2015 were available in this study.

### 3.3. Sample Preparation

Prior to brewing, the roasted coffee beans were finely ground by a coffee grinder (model GVX212, Krupps, Essen, Germany). Espresso coffee brew was then prepared from 16 g of the ground coffee to result in a volume of 60 mL using an espresso coffee-making machine (model HD8325, Philips Saeco Poemia, Bologna, Italy). This procedure involved the extraction of compounds in ground coffee using hot water (91–95 °C) and a high pressure (15 bar). One milliliter of espresso coffee brew was immediately transferred into a 10-mL headspace vial containing 400 mg of sodium chloride, which was then closed tightly by an aluminum cap that was sealed with a PTFE/silicone septum by using an electronic crimper. The vial was then vortexed for 15 s. Sodium chloride was added to facilitate the salting-out effect, thus releasing more volatile compounds from the liquid phase into the headspace (gas phase). The preparation of each coffee brew sample was repeated in triplicate.

### 3.4. SHS GC−MS

The sample vials were placed in the sample tray of a 7697A static headspace autosampler connected to a 7890B GC system and a 5977B mass spectrometer (Agilent Technologies, Palo Alto, CA, USA). The SHS GC−MS system was controlled by Agilent MassHunter GC−MS Acquisition software, version B.07.04. The extraction temperature (50, 60, 70 and 80 °C, at an equilibration time of 60 min) and the equilibration time (15, 30, 45 and 60 min, at the optimum extraction temperature) were investigated in order to select the conditions resulting in the highest total peak of volatile compounds in the coffee brew samples. Each set of conditions was repeated in triplicate. The vials were shaken at the maximum frequency of 250 times/min. The vial pressurization and injection time were set at 15 psi and 0.5 min, respectively. The sampled headspace containing volatile compounds was directly introduced into a GC−MS system. The GC injector temperature and split ratio were set at 230 °C and 5:1, respectively. The volatile compounds were separated on a DB−WAX capillary column (60 m × 0.25 mm i.d. × 0.25 µm film thickness, J & W Scientific Inc., Folsom, CA, USA). Ultrahigh purity helium was used as a carrier gas, with a constant flow rate (1.4 mL/min) corresponding to an average linear velocity of 30 cm/s. The oven temperature program was as follows: initial temperature of 40 °C, heated to 200 °C at a rate of 3 °C/min, increased to 230 °C at a rate of 50 °C/min, and then held for 2 min. The temperatures of the ion source and quadrupole were set at 230 and 150 °C, respectively. The magnitude of the electron ionization (EI) voltage was 70 eV. Mass spectra were acquired over a scan range of 35 to 300 amu with a scan speed of 1562 amu/s. For method validation, a precision test was performed by analyzing six vials of samples from the same coffee brewing. The relative standard deviation of the total peak areas of volatile compounds was evaluated.

### 3.5. Identification of Volatile Compounds

Data acquisition and peak integration were managed using Agilent MassHunter Qualitative Analysis software, version B.07.02. Data processing was further performed using Microsoft Excel 2013. The tentative identification of the volatile compounds in the coffee brew samples was achieved by comparing both their mass spectra and the linear retention index (*LRI*) with those contained in the NIST 14 database. The criteria for compound identification required a mass spectrum matching score of ≥70 and an *LRI* difference of ≤20 units between the calculated *LRI* and the database values for the same stationary phase. In this study, DB−WAX, a polar stationary phase, was applied. The *LRI* value was determined for each peak of a coffee brew sample relative to *n-*alkane retention time data obtained from the injection of two series of *n*-alkane standards (C_8_ to C_20_ and C_10_ to C_40_) using the same experimental conditions as those applied for the sample separation. After the temperature-programmed separation was performed, *LRI* values were calculated according to [38,39]:

(1) $$LRI=100n+100(\frac{t_{R\left(i\right)}-t_{R\left(n\right)}}{t_{R\left(n+1\right)}-t_{R\left(n\right)}}$$

where *t_R_* is the retention time of peak *i*, and *n* and *n* + 1 are the carbon numbers of the alkane standards bracketing the peak *i*.

### 3.6. Calculation of the Percentage of the Relative Peak Area

The percentage of the relative peak area (%RPA) of a peak in each coffee sample was calculated by dividing the peak area by the total peak area of all identified peaks in each chromatogram. The total ion chromatogram (TIC) of each sample was used for peak area integration. 

### 3.7. Data Analysis

A dataset consisting of a 36 × 78 matrix was generated. The rows and columns of the matrix represented 36 coffee samples from four subgroups (E13, NE13, E15 and NE15) and 78 volatile compounds (variables), respectively. The %RPAs were standardized before any chemometric processing. The chemometric techniques used in this work were hierarchical cluster analysis (HCA), principal component analysis (PCA), linear discriminant analysis (LDA) and iterative reformulation. Most of the chemometric methods were performed using MATLAB software, version R2018a, while HCA was processed using XLSTAT 2018 software. In addition, the analysis of variance (one-way ANOVA), T-statistics and fold-change of compounds were calculated by using Microsoft Excel 2013.

## 4. Conclusions

Volatile compound profiles and the discriminant markers of elephant dung coffee were obtained by using SHS GC-MS and chemometrics. Seventy-eight tentative compounds belonging to 13 chemical classes were identified. Four discriminant markers of elephant dung coffee, including 3-methyl-1-butanol, 2-methyl-1-butanol, 2-furfurylfuran and 3-penten-2-one, were proposed according to the T-statistics and iterative reformulation approaches. The developed methods may be useful for elephant dung coffee authentication and quality control. Furthermore, comparative sensory evaluation of elephant dung coffee has not been reported and can be subjected to investigation in the future. However, the data related to the odor description of the studied compounds were provided in Table 1.

## Figures and Tables

**Table 1:** Tentative volatile compounds of elephant dung coffee brew obtained by SHS GC-MS.

Peak No.	RT (min)	Tentative Compound	CAS No.	*LRI*		Odor Description ^c^
				Exp ^a^	Database ^b^	
					Mean ± SD (*n*)	
		**Alcohols**				
13	9.70	2-Butanol	78-92-2	1028	1025 ± 11 (104)	Fruity
14	10.17	2-Methyl-3-buten-2-ol	115-18-4	1043	1038 ± 11 (48)	Herby
28	16.61	2-Methyl-1-butanol	137-32-6	1213	1208 ± 5 (128)	Roasted
29	16.66	3-Methyl-1-butanol	123-51-3	1214	1209 ± 9 (376)	Fermented
32	18.41	3-Methyl-3-buten-1-ol	763-32-6	1255	1248 ± 8 (72)	Fruity
38	21.51	3-Methyl-2-buten-1-ol	556-82-1	1327	1320 ± 8 (48)	Fruity
		**Aldehydes**				
7	6.87	2-Methylbutanal	96-17-3	917	914 ± 8 (126)	Chocolatey
8	6.94	3-Methylbutanal	590-86-3	920	918 ± 7 (202)	Aldehydic
18	11.49	Hexanal	66-25-1	1084	1083 ± 8 (553)	Green
19	12.00	2-Methyl-2-butenal	1115-11-3	1100	1095 ± 7 (37)	Green
57	30.04	Benzaldehyde	100-52-7	1529	1520 ± 14 (471)	Fruity
		**Carboxylic acid**				
50	27.07	Acetic acid	64-19-7	1457	1449 ± 13 (380)	Acidic
		**Esters**				
1	4.82	Methyl formate	107-31-3	<800	768 ± 11 (6)	Fruity
4	5.51	Methyl acetate	79-20-9	828	828 ± 6 (63)	Ethereal
58	30.28	1-Hydroxy-2-butanone acetate	1575-57-1	1535	1536 ± 17 (13)	-
		**Furans**				
2	5.10	Furan	110-00-9	<800	799 ± 6 (22)	Ethereal
5	6.09	2-Methylfuran	534-22-5	871	869 ± 7 (52)	Chocolatey
9	7.72	2,5-Dimethylfuran	625-86-5	954	939 ± 9 (40)	Meaty
31	17.76	Furfuryl methyl ether	13679-46-4	1240	1247 ± 5 (12)	Coffee
48	26.76	*cis*-Linalool oxide	5989-33-3	1450	1444 ± 19 (175)	Earthy
51	27.47	Furfural	98-01-1	1467	1461 ± 11 (289)	Bready
53	27.95	*cis*-Linalol oxide	-	1478	1465 ± 20 (13)	-
56	29.26	2-Acetylfuran	1192-62-7	1510	1499 ± 10 (133)	Balsamic
59	30.47	Furfuryl acetate	623-17-6	1540	1531 ± 10 (40)	Fruity
60	32.02	5-Methylfurfural	620-02-0	1578	1570 ± 10 (146)	Caramelly
61	32.16	2-Propionylfuran	3194-15-8	1581	1563 ± 3 (22)	-
62	32.93	Furfuryl propionate	623-19-8	1601	1601 ± 18 (16)	Fruity
63	33.44	2-Furfurylfuran	1197-40-6	1614	1632 ± 5 (10)	Roasted
65	33.73	2-Acetyl-5-methylfuran	1193-79-9	1621	1606 ± 10 (26)	Nutty
68	34.31	γ-Butyrolactone	96-48-0	1636	1632 ± 15 (109)	Creamy
70	35.45	2-Furanmethanol	98-00-0	1666	1660 ± 9 (154)	Bready
74	47.04	Furfuryl ether	4437-22-3	1990	1986 ± 9 (3)	Coffee
		**Furanone**				
33	18.99	Dihydro-2-methyl-3(2*H*)-furanone	3188-00-9	1,268	1,268 ± 15 (52)	Bready
		**Ketones**				
3	5.33	Acetone	67-64-1	816	819 ± 6 (114)	Solvent
6	6.61	2-Butanone	78-93-3	905	907 ± 11 (109)	Ethereal
10	8.24	2,3-Butanedione	431-03-8	976	979 ± 10 (241)	Buttery
11	8.33	3-Pentanone	96-22-0	980	980 ± 6 (34)	Ethereal
15	10.52	3-Hexanone	589-38-8	1054	1053 ± 5 (45)	Fruity
16	10.66	2,3-Pentanedione	600-14-6	1058	1058 ± 9 (143)	Buttery
20	13.15	2,3-Hexanedione	3848-24-6	1129	1136 ± 2 (9)	Buttery
21	13.29	3-Penten-2-one	625-33-2	1132	1128 ± 9 (36)	Fruity
22	13.48	3,4-Hexanedione	4437-51-8	1137	1143 ± 8 (11)	Buttery
36	20.01	3-Hydroxybutanone	513-86-0	1292	1284 ± 12 (240)	Buttery
37	20.61	1-Hydroxy-2-propanone	116-09-6	1306	1303 ± 12 (62)	Caramelly
43	23.63	2-Methyl-2-cyclopenten-1-one	1120-73-6	1376	1367 ± 12 (30)	-
55	29.09	3,4,4-Trimethyl-2-cyclopenten-1-one	30434-65-2	1506	1498 ± N/A (1)	-
		**Oxazoles**				
24	14.21	4,5-Dimethyloxazole	20662-83-3	1155	1148 ± 8 (16)	-
27	16.25	Trimethyloxazole	20662-84-4	1205	1197 ± 6 (31)	Nutty
		**Phenolic compounds**				
72	42.84	Guaiacol	90-05-1	1867	1861 ± 13 (207)	Phenolic
75	47.76	Phenol	108-95-2	2013	2000 ± 15 (170)	Phenolic
77	48.62	4-Ethylguaiacol	2785-89-9	2040	2032 ± 12 (85)	Spicy
78	50.50	*p*-Cresol	106-44-5	2100	2080 ± 12 (105)	Phenolic
		**Pyrazines**				
30	16.84	Pyrazine	290-37-9	1218	1212 ± 12 (59)	Nutty
34	19.17	Methylpyrazine	109-08-0	1273	1266 ± 10 (129)	Nutty
39	21.63	2,5-Dimethylpyrazine	123-32-0	1330	1320 ± 11 (130)	Chocolatey
40	21.90	2,6-Dimethylpyrazine	108-50-9	1336	1328 ± 11 (125)	Chocolatey
41	22.12	Ethylpyrazine	13925-00-3	1341	1337 ± 12 (89)	Nutty
42	22.67	2,3-Dimethylpyrazine	5910-89-4	1354	1343 ± 10 (94)	Nutty
44	24.35	2-Ethyl-6-methylpyrazine	13925-03-6	1393	1386 ± 11 (72)	Potato
45	24.63	2-Ethyl-5-methylpyrazine	13360-64-0	1399	1387 ± 10 (76)	-
46	25.18	2-Ethyl-3-methylpyrazine	15707-23-0	1412	1407 ± 9 (52)	Nutty
47	26.42	2,6-Diethylpyrazine	13067-27-1	1442	1444 ± 15 (27)	-
49	26.88	2-Ethyl-3,5-dimethylpyrazine	13925-07-0	1453	1455 ± 9 (91)	Nutty
52	27.60	5-Ethyl-2,3-dimethylpyrazine	15707-34-3	1470	1460 ± 13 (9)	Burnt
54	28.93	3,5-Diethyl-2-methylpyrazine	18138-05-1	1502	1496 ± 7 (26)	Nutty
67	34.22	5-Methyl-6,7-dihydro-(5*H*)-cyclopentapyrazine	23747-48-0	1634	1627 ± 19 (12)	Earthy
		**Pyridine**				
26	15.77	Pyridine	110-86-1	1193	1185 ± 10 (119)	Fishy
		**Pyrroles**				
23	13.65	1-Methylpyrrole	96-54-8	1141	1145 ± 8 (39)	Woody
25	15.36	1-Ethyl-1*H*-pyrrole	617-92-5	1183	1184 ± 10 (13)	-
64	33.56	1-Ethyl-2-pyrrolecarbaldehyde	2167-14-8	1617	1610 ± 0 (7)	Roasted
66	34.01	2-Formyl-1-methylpyrrole	1192-58-1	1629	1626 ± 11 (21)	Roasted
69	35.36	2-Acetyl-1-methylpyrrole	932-16-1	1663	1656 ± 5 (12)	Earthy
71	41.70	1-Furfurylpyrrole	1438-94-4	1835	1824 ± 6 (14)	Vegetable
73	46.72	2-Acetylpyrrole	1072-83-9	1981	1973 ± 12 (56)	Musty
		**Sulfur-containing compounds**				
12	9.59	Thiophene	110-02-1	1025	1025 ± 6 (36)	Sulfurous
17	11.25	Dimethyl disulfide	624-92-0	1077	1077 ± 8 (145)	Sulfurous
35	19.79	4-Methylthiazole	693-95-8	1287	1282 ± 9 (24)	Nutty

^a^ Exp = Experimental linear retention indices calculated using *n*-alkane standards on a DB-WAX column; ^b^ Database = Linear retention indices obtained from NIST 14 database; ^c^ Odor description obtained from http://www.thegoodscentscompany.com [35].

**Table 2:** The LDA classification results of coffee samples from different groups: elephant dung coffee (E) and control (NE) samples produced in 2013 and 2015.

Sample	Predicted Group Membership				Correct Classification (%)
	E13	E15	NE13	NE15	
E13	6	3	0	0	67
E15	1	8	0	0	89
NE13	0	0	9	0	100
NE15	0	0	0	9	100
Predictive ability (%)					89

Remark. E13 = Elephant dung coffee produced in 2013, E15 = Elephant dung coffee produced in 2015, NE13 = Control sample of elephant dung coffee produced in 2013, and NE15 = Control sample of elephant dung coffee produced in 2015.

**Table 3:** Iterative reformulation results and statistical data of selected volatile compounds from elephant dung coffee and controls.

Peak No.	Volatile Compound	Frequency of Selection (Times)	*t*-Stat	*p*-Value	Fold-Change
29	3-Methyl-1-butanol	100	−18.5	<0.01	−2.71
28	2-Methyl-1-butanol	100	−14.6	<0.01	−2.19
21	3-Penten-2-one	99	−13.0	<0.01	−1.45
63	2-Furfurylfuran	100	−12.5	<0.01	−2.37
31	Furfuryl methyl ether	73	−9.91	<0.01	−1.72
43	2-Methyl-2-cyclopenten-1-one	18	−9.18	<0.01	−1.74
7	2-Methylbutanal	5	8.96	<0.01	1.55
8	3-Methylbutanal	3	8.30	< 0.01	1.61

## Supplementary Materials

The following are available online, Table S1: The relative standard deviations (RSDs) of the percentage of the relative peak areas (%RPAs) of volatile compounds obtained from SHS GC-MS, and Table S2: Statistical data of volatile compounds obtained from elephant dung coffee and control samples.
