# Supplementary material for: Identification of Volatile Compounds and Selection of Discriminant Markers for Elephant Dung Coffee Using Static Headspace Gas Chromatography—Mass Spectrometry and Chemometrics
Source: Molecules. 2018 Jul 31;23(8):1910. doi: 10.3390/molecules23081910 (PMC6222725; doi:10.3390/molecules23081910)
Supplement: Supplementary file 1 [file molecules-23-01910-s001.pdf]

Supplementary Materials

Table S1. The relative standard deviations (RSDs) of the percentage of the relative peak areas (%RPAs) of volatile compounds obtained from SHS GC–MS.

Peak no.	Tentative compound	%RPA							
		Rep1	Rep2	Rep3	Rep4	Rep5	Rep6	Mean	**RSD (%)**
1	Methyl formate	0.10	0.09	0.09	0.10	0.10	0.09	0.09	**7.6**
2	Furan	0.99	0.94	0.98	0.99	0.98	0.99	0.98	**1.9**
3	Acetone	13.03	12.22	12.17	13.29	12.78	12.60	12.68	**3.5**
4	Methyl acetate	2.39	1.98	2.15	2.12	2.05	1.90	2.10	**8.0**
5	2-Methylfuran	0.97	1.07	1.04	0.82	0.89	0.89	0.95	**10**
6	2-Butanone	2.75	2.64	2.58	2.69	2.51	2.44	2.60	**4.4**
7	2-Methylbutanal	7.70	7.65	7.68	8.05	8.10	8.14	7.89	**3.0**
8	3-Methylbutanal	2.60	2.66	2.63	2.95	2.78	2.78	2.73	**4.7**
9	2,5-Dimethylfuran	0.08	0.08	0.09	0.08	0.08	0.09	0.08	**3.3**
10	2,3-Butanedione	1.08	1.07	1.03	1.09	1.12	1.09	1.08	**2.7**
11	3-Pentanone	0.58	0.57	0.56	0.54	0.55	0.54	0.56	**3.3**
12	Thiophene	0.05	0.05	0.06	0.05	0.06	0.05	0.05	**6.2**
13	2-Butanol	0.05	0.05	0.05	0.05	0.06	0.05	0.05	**7.6**
14	2-Methyl-3-buten-2-ol	0.37	0.35	0.35	0.40	0.39	0.38	0.37	**5.2**
15	3-Hexanone	0.06	0.06	0.06	0.06	0.07	0.08	0.07	**12**
16	2,3-Pentanedione	0.77	0.80	0.76	0.81	0.88	0.83	0.81	**5.7**
17	Dimethyl disulfide	0.05	0.05	0.05	0.05	0.06	0.06	0.05	**12**
18	Hexanal	0.09	0.10	0.11	0.08	0.09	0.10	0.09	**11**
19	2-Methyl-2-butenal	0.08	0.08	0.09	0.08	0.08	0.08	0.08	**3.9**
20	2,3-Hexanedione	0.06	0.07	0.08	0.05	0.07	0.07	0.07	**12**
21	3-Penten-2-one	0.07	0.07	0.07	0.06	0.07	0.07	0.07	**5.9**
22	3,4-Hexanedione	0.04	0.05	0.05	0.04	0.04	0.04	0.04	**11**
23	1-Methylpyrrole	0.16	0.18	0.17	0.14	0.15	0.15	0.16	**9.6**
24	4,5-Dimethyloxazole	0.08	0.08	0.09	0.06	0.07	0.07	0.07	**13**
25	1-Ethyl-1H-pyrrole	0.06	0.07	0.07	0.05	0.06	0.07	0.06	**11**
26	Pyridine	1.40	1.47	1.52	1.23	1.41	1.53	1.43	**7.8**
27	Trimethyloxazole	0.10	0.11	0.11	0.09	0.10	0.11	0.10	**9.2**
28	2-Methyl-1-butanol	0.08	0.09	0.09	0.06	0.08	0.07	0.08	**14**
29	3-Methyl-1-butanol	0.10	0.12	0.11	0.10	0.10	0.09	0.10	**8.8**
30	Pyrazine	0.57	0.55	0.52	0.56	0.52	0.51	0.54	**4.9**
31	Furfuryl methyl ether	0.29	0.29	0.30	0.20	0.25	0.26	0.26	**14**
32	3-Methyl-3-buten-1-ol	0.28	0.28	0.29	0.27	0.23	0.29	0.27	**7.4**
33	Dihydro-2-methyl-3(2H)-furanone	1.32	1.18	1.12	1.27	1.19	1.17	1.21	**6.0**
34	Methylpyrazine	6.21	5.74	5.69	5.68	5.57	5.51	5.73	**4.3**
35	4-Methylthiazole	0.10	0.10	0.10	0.07	0.07	0.08	0.09	**15**
36	3-Hydroxybutanone	0.17	0.16	0.15	0.15	0.14	0.16	0.16	**6.3**
37	1-Hydroxy-2-propanone	0.30	0.35	0.32	0.40	0.37	0.41	0.36	**13**
38	3-Methyl-2-buten-1-ol	0.06	0.06	0.05	0.06	0.06	0.06	0.06	**5.8**
39	2,5-Dimethylpyrazine	3.14	3.04	3.02	2.85	2.91	2.90	2.98	**3.7**
40	2,6-Dimethylpyrazine	3.23	2.95	3.11	2.83	2.97	2.95	3.01	**4.6**
41	Ethylpyrazine	2.56	2.27	2.43	2.30	2.34	2.34	2.37	**4.5**
42	2,3-Dimethylpyrazine	0.67	0.65	0.65	0.58	0.60	0.60	0.62	**5.7**
43	2-Methyl-2-cyclopenten-1-one	0.12	0.11	0.11	0.09	0.12	0.12	0.11	**11**

44	2-Ethyl-6-methylpyrazine	2.58	2.74	2.77	2.31	2.58	2.55	2.58	**6.4**
45	2-Ethyl-5-methylpyrazine	1.88	2.00	2.10	1.83	1.90	1.90	1.94	**4.8**
46	2-Ethyl-3-methylpyrazine	2.28	2.26	2.25	2.03	2.15	2.16	2.19	**4.2**
47	2,6-Diethylpyrazine	0.10	0.09	0.11	0.07	0.08	0.09	0.09	**13**
48	*cis*-Linalool oxide	0.17	0.20	0.20	0.15	0.18	0.18	0.18	**11**
49	2-Ethyl-3,5-dimethylpyrazine	2.18	2.32	2.38	1.96	2.24	2.30	2.23	**6.6**
50	Acetic acid	2.23	2.23	2.28	2.50	2.24	2.27	2.29	**4.6**
51	Furfural	7.08	6.69	6.59	6.94	6.76	6.68	6.79	**2.7**
52	5-Ethyl-2,3-dimethylpyrazine	0.47	0.51	0.51	0.44	0.49	0.51	0.49	**5.9**
53	*cis*-Linalol oxide	0.16	0.17	0.18	0.14	0.17	0.18	0.17	**9.2**
54	3,5-Diethyl-2-methylpyrazine	0.31	0.36	0.36	0.34	0.41	0.43	0.37	**11**
55	3,4,4-Trimethyl-2-cyclopenten-1-one	0.07	0.07	0.08	0.07	0.10	0.09	0.08	**12**
56	2-Acetylfuran	1.92	1.78	1.77	1.70	1.64	1.61	1.74	**6.4**
57	Benzaldehyde	0.48	0.48	0.48	0.46	0.50	0.52	0.49	**4.3**
58	1-Hydroxy-2-butanone acetate	0.33	0.32	0.32	0.31	0.27	0.28	0.31	**8.1**
59	Furfuryl acetate	0.88	0.95	0.99	0.90	1.01	1.08	0.97	**7.7**
60	5-Methylfurfural	5.81	5.62	5.57	5.74	5.82	5.77	5.72	**1.8**
61	2-Propionylfuran	0.48	0.48	0.48	0.40	0.42	0.43	0.45	**8.6**
62	Furfuryl propionate	0.10	0.13	0.11	0.11	0.12	0.13	0.12	**9.3**
63	2-Furfurylfuran	0.13	0.15	0.15	0.13	0.16	0.15	0.14	**9.2**
64	1-Ethyl-2-pyrrolecarbaldehyde	0.10	0.10	0.10	0.08	0.10	0.10	0.10	**7.9**
65	2-Acetyl-5-methylfuran	0.12	0.11	0.12	0.08	0.11	0.11	0.11	**13**
66	2-Formyl-1-methylpyrrole	0.69	0.67	0.69	0.64	0.68	0.68	0.67	**2.8**
67	5-Methyl-6,7-dihydro-(5H)-cyclopentapyrazine	0.09	0.08	0.09	0.08	0.08	0.08	0.08	**8.8**
68	γ-Butyrolactone	0.35	0.38	0.34	0.36	0.34	0.36	0.36	**4.2**
69	2-Acetyl-1-methylpyrrole	0.32	0.32	0.35	0.26	0.29	0.32	0.31	**10**
70	2-Furanmethanol	12.60	15.00	14.60	15.41	14.83	15.00	14.57	**6.9**
71	1-Furfurylpyrrole	0.24	0.29	0.30	0.24	0.26	0.27	0.27	**8.9**
72	Guaiacol	0.09	0.09	0.10	0.09	0.11	0.11	0.10	**9.6**
73	2-Acetylpyrrole	0.20	0.22	0.22	0.18	0.19	0.22	0.21	**7.4**
74	Furfuryl ether	0.14	0.16	0.18	0.16	0.18	0.19	0.17	**11**
75	Phenol	0.22	0.23	0.23	0.15	0.20	0.19	0.20	**15**
76	1H-Pyrrole-2-carboxaldehyde	0.15	0.15	0.15	0.16	0.17	0.17	0.16	**6.0**
77	4-Ethylguaiacol	0.05	0.05	0.05	0.04	0.05	0.05	0.05	**11**
78	*p*-Cresol	0.03	0.03	0.03	0.03	0.03	0.03	0.03	**9.7**

Table S2. Statistical data of volatile compounds obtained from elephant dung coffee and control samples.

Peak no.	Year 2013					Year 2015					Year 2013 & Year 2015				
	%RPA (*n* = 9)		Fold-change	*t*-stat	*p*-value	%RPA (*n* = 9)		Fold-change	*t*-stat	*p*-value	%RPA (*n* = 18)		Fold-change	*t*-stat	*p*-value
	E13	NE13				E15	NE15				E13 & E15	NE13 & NE15			
1	0.09 ± 0.01	0.11 ± 0.03	-1.25	-1.74	0.10	0.08 ± 0.03	0.04 ± 0.01	2.14	4.10	<0.01	0.08 ± 0.02	0.07 ± 0.04	1.15	0.97	0.34
2	0.99 ± 0.02	1.24 ± 0.11	-1.25	-6.60	<0.01	1.09 ± 0.10	1.07 ± 0.13	1.01	0.22	0.83	1.04 ± 0.09	1.16 ± 0.15	-1.12	-3.00	<0.01
3	12.35 ± 0.63	11.81 ± 0.56	1.05	1.93	0.07	12.81 ± 1.90	10.86 ± 0.69	1.18	2.89	0.01	12.58 ± 1.39	11.33 ± 0.78	1.11	3.31	<0.01
4	2.06 ± 0.13	3.13 ± 0.22	-1.52	-12.48	<0.01	3.50 ± 0.49	3.55 ± 0.20	-1.01	-0.29	0.78	2.78 ± 0.82	3.34 ± 0.30	-1.20	-2.74	<0.01
5	0.96 ± 0.10	1.83 ± 0.40	-1.90	-6.27	<0.01	1.18 ± 0.21	2.04 ± 0.45	-1.72	-5.17	<0.01	1.07 ± 0.20	1.93 ± 0.43	-1.80	-7.77	<0.01
6	2.58 ± 0.08	3.27 ± 0.17	-1.27	-10.78	<0.01	3.39 ± 0.47	3.62 ± 0.33	-1.07	-1.22	0.24	2.98 ± 0.53	3.45 ± 0.31	-1.15	-3.19	<0.01
7	7.55 ± 0.58	4.87 ± 0.65	1.55	9.22	<0.01	7.35 ± 1.45	4.76 ± 0.64	1.54	4.89	<0.01	7.45 ± 1.08	4.82 ± 0.63	1.55	8.96	<0.01
8	2.61 ± 0.24	1.69 ± 0.26	1.55	7.94	<0.01	2.30 ± 0.47	1.36 ± 0.17	1.69	5.64	<0.01	2.46 ± 0.40	1.52 ± 0.27	1.61	8.30	<0.01
9	0.09 ± 0.01	0.15 ± 0.01	-1.73	-15.88	<0.01	0.11 ± 0.01	0.12 ± 0.02	-1.08	-1.35	0.20	0.10 ± 0.01	0.13 ± 0.02	-1.37	-5.95	<0.01
10	1.07 ± 0.04	0.96 ± 0.10	1.12	3.20	<0.01	0.92 ± 0.05	0.70 ± 0.09	1.31	6.44	<0.01	0.99 ± 0.09	0.83 ± 0.16	1.20	3.82	<0.01
11	0.51 ± 0.07	0.79 ± 0.10	-1.54	-6.87	<0.01	0.74 ± 0.08	0.93 ± 0.13	-1.27	-3.76	<0.01	0.63 ± 0.14	0.86 ± 0.14	-1.38	-5.21	<0.01
12	0.06 ± 0.01	0.07 ± 0.01	-1.28	-4.62	<0.01	0.06 ± 0.01	0.08 ± 0.01	-1.27	-2.83	0.01	0.06 ± 0.01	0.08 ± 0.01	-1.27	-4.65	<0.01
13	0.05 ± 0.01	0.08 ± 0.04	-1.54	-2.44	0.03	0.06 ± 0.01	0.07 ± 0.01	-1.33	-2.86	0.01	0.05 ± 0.01	0.08 ± 0.03	-1.43	-3.58	<0.01
14	0.37 ± 0.02	0.35 ± 0.05	1.07	1.58	0.13	0.34 ± 0.02	0.25 ± 0.02	1.39	10.29	<0.01	0.36 ± 0.02	0.30 ± 0.06	1.21	3.89	<0.01
15	0.06 ± 0.01	0.09 ± 0.02	-1.46	-4.61	<0.01	0.09 ± 0.02	0.11 ± 0.01	-1.26	-2.92	0.01	0.07 ± 0.02	0.10 ± 0.02	-1.34	-4.16	<0.01
16	0.78 ± 0.06	0.79 ± 0.17	-1.02	-0.22	0.83	0.54 ± 0.06	0.39 ± 0.06	1.39	5.25	<0.01	0.66 ± 0.14	0.59 ± 0.24	1.12	1.05	0.30
17	0.09 ± 0.05	0.04 ± 0.03	1.96	2.20	0.04	0.07 ± 0.03	0.03 ± 0.01	2.38	4.02	<0.01	0.08 ± 0.04	0.04 ± 0.02	2.13	3.70	<0.01
18	0.09 ± 0.01	0.21 ± 0.06	-2.32	-5.51	<0.01	0.08 ± 0.01	0.09 ± 0.02	-1.12	-1.37	0.19	0.09 ± 0.01	0.15 ± 0.07	-1.74	-3.63	<0.01
19	0.08 ± 0.01	0.07 ± 0.01	1.19	5.04	<0.01	0.09 ± 0.02	0.08 ± 0.01	1.15	2.01	0.06	0.08 ± 0.01	0.07 ± 0.01	1.17	3.29	<0.01
20	0.08 ± 0.03	0.10 ± 0.01	-1.25	-2.26	0.04	0.07 ± 0.01	0.09 ± 0.01	-1.17	-2.27	0.04	0.08 ± 0.02	0.10 ± 0.01	-1.21	-2.96	<0.01
21	0.07 ± 0.01	0.11 ± 0.01	-1.54	-11.53	<0.01	0.08 ± 0.01	0.12 ± 0.01	-1.37	-9.67	<0.01	0.08 ± 0.01	0.11 ± 0.01	-1.45	-12.97	<0.01
22	0.05 ± 0.02	0.07 ± 0.02	-1.33	-2.42	0.03	0.04 ± 0.01	0.05 ± 0.01	-1.19	-1.28	0.22	0.05 ± 0.02	0.06 ± 0.02	-1.27	-2.30	0.03
23	0.16 ± 0.01	0.14 ± 0.04	1.18	1.91	0.07	0.21 ± 0.03	0.45 ± 0.08	-2.17	-8.21	<0.01	0.18 ± 0.03	0.30 ± 0.18	-1.60	-2.62	0.01
24	0.08 ± 0.02	0.11 ± 0.01	-1.31	-3.96	<0.01	0.08 ± 0.01	0.12 ± 0.02	-1.43	-5.50	<0.01	0.08 ± 0.01	0.11 ± 0.01	-1.37	-6.78	<0.01
25	0.07 ± 0.01	0.08 ± 0.04	-1.18	-0.93	0.37	0.06 ± 0.02	0.10 ± 0.02	-1.80	-5.47	<0.01	0.06 ± 0.02	0.09 ± 0.03	-1.46	-3.51	<0.01
26	1.29 ± 0.20	1.37 ± 0.37	-1.06	-0.55	0.59	1.88 ± 0.24	7.05 ± 1.55	-3.74	-9.89	<0.01	1.59 ± 0.37	4.21 ± 3.12	-2.65	-3.54	<0.01
27	0.09 ± 0.02	0.12 ± 0.02	-1.34	-3.27	<0.01	0.09 ± 0.02	0.16 ± 0.05	-1.69	-3.40	<0.01	0.09 ± 0.02	0.14 ± 0.04	-1.52	-4.23	<0.01
28	0.09 ± 0.02	0.19 ± 0.01	-2.16	-14.11	<0.01	0.11 ± 0.01	0.24 ± 0.02	-2.22	-16.10	<0.01	0.10 ± 0.02	0.22 ± 0.03	-2.19	-14.60	<0.01
29	0.11 ± 0.02	0.36 ± 0.02	-3.14	-25.97	<0.01	0.18 ± 0.02	0.43 ± 0.03	-2.44	-25.56	<0.01	0.15 ± 0.04	0.40 ± 0.04	-2.71	-18.53	<0.01
30	0.54 ± 0.02	0.53 ± 0.02	1.02	0.97	0.35	0.50 ± 0.03	0.66 ± 0.04	-1.34	-9.46	<0.01	0.52 ± 0.03	0.60 ± 0.08	-1.15	-4.00	<0.01
31	0.27 ± 0.05	0.60 ± 0.05	-2.19	-13.89	<0.01	0.41 ± 0.04	0.58 ± 0.08	-1.40	-5.65	<0.01	0.34 ± 0.08	0.59 ± 0.06	-1.72	-9.91	<0.01
32	0.31 ± 0.05	0.58 ± 0.08	-1.89	-8.89	<0.01	0.26 ± 0.05	0.26 ± 0.04	1.01	0.09	0.93	0.29 ± 0.05	0.42 ± 0.17	-1.47	-3.15	<0.01
33	1.16 ± 0.09	1.71 ± 0.21	-1.48	-7.11	<0.01	1.32 ± 0.11	0.94 ± 0.11	1.41	7.26	<0.01	1.24 ± 0.13	1.32 ± 0.43	-1.07	-0.79	0.43
34	5.57 ± 0.28	4.51 ± 0.10	1.23	10.71	<0.01	5.00 ± 0.13	5.23 ± 0.29	-1.05	-2.22	0.04	5.28 ± 0.36	4.87 ± 0.42	1.08	3.12	<0.01
35	0.10 ± 0.02	0.11 ± 0.01	-1.09	-1.19	0.25	0.07 ± 0.01	0.10 ± 0.03	-1.45	-3.51	<0.01	0.08 ± 0.02	0.11 ± 0.02	-1.24	-3.16	<0.01
36	0.20 ± 0.06	0.21 ± 0.05	-1.08	-0.55	0.59	0.15 ± 0.03	0.13 ± 0.04	1.14	1.20	0.25	0.18 ± 0.05	0.17 ± 0.06	1.01	0.09	0.93
37	0.39 ± 0.06	0.38 ± 0.08	1.03	0.32	0.76	0.23 ± 0.07	0.12 ± 0.03	1.88	3.98	<0.01	0.31 ± 0.11	0.25 ± 0.15	1.23	1.37	0.18

38	0.06 ± 0.01	0.06 ± 0.01	1.02	0.28	0.79	0.06 ± 0.01	0.05 ± 0.02	1.42	2.87	0.01	0.06 ± 0.01	0.05 ± 0.02	1.19	2.40	0.02
39	2.87 ± 0.17	1.80 ± 0.07	1.59	17.39	<0.01	2.61 ± 0.12	2.50 ± 0.13	1.04	1.76	0.10	2.74 ± 0.20	2.15 ± 0.37	1.27	5.89	<0.01
40	2.96 ± 0.11	2.00 ± 0.05	1.48	24.22	<0.01	2.73 ± 0.26	2.60 ± 0.19	1.05	1.25	0.23	2.85 ± 0.23	2.30 ± 0.34	1.24	5.74	<0.01
41	2.29 ± 0.14	1.75 ± 0.07	1.31	10.04	<0.01	1.99 ± 0.07	2.30 ± 0.12	-1.16	-6.69	<0.01	2.14 ± 0.19	2.03 ± 0.30	1.06	1.37	0.18
42	0.62 ± 0.03	0.52 ± 0.01	1.17	9.57	<0.01	0.54 ± 0.03	0.71 ± 0.05	-1.30	-8.53	<0.01	0.58 ± 0.05	0.62 ± 0.10	-1.06	-1.41	0.17
43	0.11 ± 0.02	0.20 ± 0.03	-1.73	-6.50	<0.01	0.14 ± 0.01	0.24 ± 0.03	-1.74	-9.24	<0.01	0.13 ± 0.02	0.22 ± 0.04	-1.74	-9.18	<0.01
44	2.58 ± 0.13	1.50 ± 0.05	1.72	23.59	<0.01	2.07 ± 0.42	2.62 ± 0.24	-1.27	-3.41	<0.01	2.33 ± 0.40	2.06 ± 0.60	1.13	1.55	0.13
45	1.86 ± 0.14	1.15 ± 0.04	1.62	15.05	<0.01	1.69 ± 0.12	1.82 ± 0.11	-1.08	-2.45	0.03	1.78 ± 0.15	1.49 ± 0.36	1.20	3.21	<0.01
46	2.11 ± 0.14	1.28 ± 0.06	1.64	16.73	<0.01	1.78 ± 0.11	2.25 ± 0.06	-1.27	-11.29	<0.01	1.94 ± 0.21	1.77 ± 0.50	1.10	1.37	0.18
47	0.15 ± 0.10	0.08 ± 0.04	1.96	2.16	0.05	0.10 ± 0.09	0.15 ± 0.11	-1.56	-1.18	0.25	0.13 ± 0.09	0.11 ± 0.09	1.09	0.36	0.72
48	0.30 ± 0.21	0.15 ± 0.09	2.01	1.97	0.07	0.23 ± 0.21	0.13 ± 0.11	1.75	1.24	0.23	0.27 ± 0.21	0.14 ± 0.10	1.89	2.30	0.03
49	2.12 ± 0.22	0.86 ± 0.07	2.48	16.73	<0.01	1.55 ± 0.23	2.48 ± 0.18	-1.59	-9.45	<0.01	1.84 ± 0.36	1.67 ± 0.84	1.10	0.78	0.44
50	2.84 ± 0.83	4.39 ± 1.69	-1.55	-2.47	0.03	2.87 ± 1.17	1.00 ± 0.67	2.87	4.16	<0.01	2.85 ± 0.99	2.69 ± 2.14	1.06	0.29	0.77
51	6.52 ± 0.44	6.89 ± 0.72	-1.06	-1.32	0.20	5.92 ± 0.27	3.90 ± 0.38	1.52	13.04	<0.01	6.22 ± 0.47	5.39 ± 1.64	1.15	2.06	0.05
52	0.47 ± 0.04	0.23 ± 0.01	2.01	16.51	<0.01	0.36 ± 0.06	0.62 ± 0.18	-1.71	-3.96	<0.01	0.41 ± 0.08	0.42 ± 0.24	-1.03	-0.19	0.85
53	0.22 ± 0.11	0.13 ± 0.06	1.71	2.23	0.04	0.20 ± 0.18	0.12 ± 0.08	1.72	1.27	0.22	0.21 ± 0.14	0.12 ± 0.07	1.71	2.32	0.03
54	0.37 ± 0.05	0.14 ± 0.03	2.68	11.36	<0.01	0.22 ± 0.05	0.45 ± 0.08	-2.05	-7.71	<0.01	0.30 ± 0.09	0.30 ± 0.17	1.00	0.01	0.99
55	0.09 ± 0.02	0.14 ± 0.03	-1.45	-3.58	<0.01	0.09 ± 0.01	0.22 ± 0.03	-2.40	-12.01	<0.01	0.09 ± 0.02	0.18 ± 0.05	-1.92	-6.75	<0.01
56	1.74 ± 0.07	2.77 ± 0.06	-1.59	-33.54	<0.01	2.02 ± 0.08	1.90 ± 0.05	1.06	3.74	<0.01	1.88 ± 0.16	2.34 ± 0.45	-1.24	-4.06	<0.01
57	0.50 ± 0.03	0.43 ± 0.08	1.16	2.49	0.02	0.58 ± 0.04	0.84 ± 0.20	-1.44	-3.72	<0.01	0.54 ± 0.06	0.64 ± 0.26	-1.17	-1.51	0.14
58	0.34 ± 0.06	0.43 ± 0.07	-1.25	-2.93	<0.01	0.25 ± 0.08	0.23 ± 0.08	1.08	0.49	0.63	0.30 ± 0.08	0.33 ± 0.12	-1.11	-0.96	0.34
59	1.17 ± 0.59	2.55 ± 0.61	-2.18	-4.88	<0.01	1.13 ± 0.76	1.97 ± 1.04	-1.74	-1.95	0.07	1.15 ± 0.66	2.26 ± 0.88	-1.96	-4.27	<0.01
60	5.49 ± 0.38	6.08 ± 0.62	-1.11	-2.46	0.03	4.93 ± 0.14	2.40 ± 0.65	2.06	11.48	<0.01	5.21 ± 0.40	4.24 ± 1.99	1.23	2.03	0.05
61	0.44 ± 0.04	0.74 ± 0.07	-1.67	-11.28	<0.01	0.50 ± 0.04	0.62 ± 0.07	-1.22	-4.18	<0.01	0.47 ± 0.05	0.68 ± 0.09	-1.43	-8.24	<0.01
62	0.15 ± 0.08	0.30 ± 0.07	-2.00	-4.27	<0.01	0.12 ± 0.04	0.23 ± 0.08	-1.87	-3.44	<0.01	0.14 ± 0.06	0.26 ± 0.08	-1.94	-5.25	<0.01
63	0.15 ± 0.04	0.37 ± 0.06	-2.51	-8.98	<0.01	0.15 ± 0.01	0.34 ± 0.06	-2.23	-8.76	<0.01	0.15 ± 0.03	0.36 ± 0.06	-2.37	-12.45	<0.01
64	0.11 ± 0.02	0.12 ± 0.01	-1.08	-1.15	0.27	0.12 ± 0.02	0.13 ± 0.01	-1.06	-0.81	0.43	0.12 ± 0.02	0.13 ± 0.01	-1.07	-1.36	0.18
65	0.11 ± 0.01	0.18 ± 0.02	-1.61	-10.04	<0.01	0.13 ± 0.02	0.14 ± 0.03	-1.08	-0.85	0.41	0.12 ± 0.02	0.16 ± 0.03	-1.32	-4.61	<0.01
66	0.67 ± 0.02	0.66 ± 0.03	1.01	0.78	0.45	0.72 ± 0.04	0.73 ± 0.04	-1.01	-0.39	0.70	0.70 ± 0.04	0.70 ± 0.05	1.00	0.04	0.97
67	0.12 ± 0.05	0.06 ± 0.02	1.85	2.88	0.01	0.06 ± 0.04	0.12 ± 0.09	-1.98	-1.87	0.08	0.09 ± 0.05	0.09 ± 0.07	-1.03	-0.13	0.89
68	0.35 ± 0.02	0.48 ± 0.10	-1.37	-3.71	<0.01	0.39 ± 0.07	0.57 ± 0.08	-1.47	-5.32	<0.01	0.37 ± 0.05	0.53 ± 0.10	-1.42	-5.88	<0.01
69	0.33 ± 0.04	0.41 ± 0.07	-1.25	-2.93	<0.01	0.31 ± 0.06	0.55 ± 0.03	-1.75	-10.04	<0.01	0.32 ± 0.05	0.48 ± 0.09	-1.49	-6.58	<0.01
70	15.23 ± 0.36	16.51 ± 0.99	-1.08	-3.62	<0.01	16.53 ± 1.54	15.99 ± 1.23	1.03	0.83	0.42	15.88 ± 1.27	16.25 ± 1.12	-1.02	-0.91	0.37
71	0.28 ± 0.02	0.26 ± 0.05	1.07	1.08	0.30	0.24 ± 0.06	0.37 ± 0.04	-1.52	-5.38	<0.01	0.26 ± 0.05	0.31 ± 0.07	-1.21	-2.68	0.01
72	0.14 ± 0.10	0.17 ± 0.05	-1.15	-0.58	0.57	0.12 ± 0.09	0.27 ± 0.14	-2.15	-2.51	0.02	0.13 ± 0.09	0.22 ± 0.12	-1.61	-2.33	0.03
73	0.27 ± 0.10	0.29 ± 0.06	-1.05	-0.34	0.74	0.22 ± 0.12	0.28 ± 0.11	-1.29	-1.13	0.27	0.25 ± 0.11	0.28 ± 0.09	-1.15	-1.13	0.27
74	0.20 ± 0.10	0.43 ± 0.09	-2.17	-5.20	<0.01	0.17 ± 0.09	0.40 ± 0.11	-2.40	-4.99	<0.01	0.18 ± 0.10	0.42 ± 0.10	-2.28	-7.32	<0.01
75	0.25 ± 0.07	0.32 ± 0.07	-1.29	-2.09	0.05	0.29 ± 0.06	0.50 ± 0.11	-1.75	-5.19	<0.01	0.27 ± 0.07	0.41 ± 0.13	-1.53	-4.19	<0.01
76	0.18 ± 0.04	0.18 ± 0.04	1.03	0.28	0.78	0.17 ± 0.06	0.10 ± 0.03	1.74	3.31	<0.01	0.18 ± 0.05	0.14 ± 0.05	1.28	2.22	0.03
77	0.07 ± 0.04	0.09 ± 0.03	-1.35	-1.37	0.19	0.07 ± 0.05	0.13 ± 0.07	-1.84	-2.00	0.06	0.07 ± 0.05	0.11 ± 0.06	-1.60	-2.42	0.02
78	0.04 ± 0.02	0.05 ± 0.02	-1.18	-0.91	0.38	0.04 ± 0.02	0.04 ± 0.01	-1.03	-0.14	0.89	0.04 ± 0.02	0.05 ± 0.01	-1.11	-0.79	0.43

%RPAs were reported as Mean ± SD, E13 = Elephant dung coffee produced in 2013, E15 = Elephant dung coffee produced in 2015, NE13 = Control sample of elephant dung coffee produced in 2013 and NE15 = Control sample of elephant dung coffee produced in 2015.
